# Admissions with airway emergencies in the ICU at a tertiary referral centre

**DOI:** 10.1186/cc14285

**Published:** 2015-03-16

**Authors:** M Gresoiu, M Singer

**Affiliations:** 1 University College London Hospital, London, UK

## Introduction

As a tertiary referral centre for ENT and maxillo-facial surgery, our ICU receives complex elective and emergency cases. The frequency, aetiology and outcomes of airway emergencies are poorly described. Understanding these factors is key to improving management.

## Methods

We conducted a retrospective review of the ICU electronic patient database examining unplanned admissions with airway emergencies between December 13 and November 14. Data on demographics, aetiology of airway obstruction (including postprocedural), APACHE II score, therapeutic intervention(s) administered, and outcomes were collected.

## Results

Of 1,516 unplanned admissions, airway emergencies represented 6.3% (96 patients) of whom 40 (41.7%) had malignancy (26 maxillo-facial/trachea, three pulmonary, four haematological, seven other) and 24 infection (abscesses, epiglottitis, Ludwig's angina). Referring specialties were maxillo-facial surgery (*n *= 34), internal medicine (*n *= 25), ENT (*n *= 21) and other surgical specialties (*n *= 16). Thirteen patients had complications post bronchoscopy (vocal cord palsy, need for NIV or intubation), one post microlaryngoscopy, and 20 were admitted after difficult intubation. Eighteen were admitted post drainage of abscess (dental, retropharyngeal) and seven for observation for epiglottitis. Thirteen patients had stridor (three tracheal stenosis, one vocal cord cyst, one post CVA, four post vocal cord palsy, one post oesophagoscopy, one post thyroidectomy, two post decannulation). Seven were admitted after emergency tracheostomy, one after blocked tracheostomy, one after emergency laryngectomy, six post bleeding (epistaxis, haemoptysis, bleed form laryngectomy site), and four post evacuation haematoma. Three were admitted following anaphylaxis/ angioedema and one after laryngospasm. Twenty-nine patients required medical management only (for example, steroids, nebulisers, and so forth), 25 were extubated post difficult intubation and six needed haemostasis control. Ten (nine surgical) tracheostomies were performed during their ICU stay. Sixteen patients died in hospital, of whom five were in the ICU at the time; 14 of these had an underlying malignancy. Twenty-three patients deteriorated during their ICU stay including HAP (*n *= 3), bleeding from airway (*n *= 3), PEA arrest (*n *= 1), airway swelling (*n *= 2), blocked laryngectomy (*n *= 1), and tracheostomy dislodgement (*n *= 1).

## Conclusion

Marked variation was seen in the type and aetiology of airway emergencies admitted to the ICU. A broad training programme is thus required to offer wide-ranging awareness of potential problems, communication (including an emergency airway plan), and acute management.

